# Photocalorespirometry (Photo-CR): A Novel Method for Access to Photosynthetic Energy Conversion Efficiency

**DOI:** 10.1038/s41598-019-45296-8

**Published:** 2019-06-26

**Authors:** Thomas Maskow, Anne Rothe, Torsten Jakob, Sven Paufler, Christian Wilhelm

**Affiliations:** 1 UFZ - Helmholtz Centre for Environmental Research, Dept. Environmental Microbiology, Leipzig, Permoserstr. 15, D-04318 Leipzig, Germany; 2 University of Leipzig, Institute of Biology, Johannisallee 21-23, D-04103 Leipzig, Germany

**Keywords:** Bioenergetics, Environmental impact, Carbon capture and storage

## Abstract

One key parameter for assessing the CO_2_ fixation in aquatic ecosystems but also for the productivity of photobioreactors is the energy conversion efficiency (PE) by the photosynthetic apparatus. PE strictly depends on a range of different fluctuating environmental conditions and is therefore highly variable. PE is the result of complex metabolic control. At the moment PE can only be determined indirectly. Furthermore, the currently available techniques either capture only short time processes, thus reflecting only parts of the photosynthetic engine, or quantify the total process but only with limited time resolution. To close this gap, we suggest for the first time the direct measurement of the fixed energy combined with respirometry, called photocalorespirometry (Photo-CR). The proof of the principle of Photo-CR was established with the microalga *Chlamydomonas reinhardtii*. The simultaneous measurement of oxygen production and energy fixation provides an calorespirometric ratio of −(437.9 ± 0.7) kJ mol^−1^ under low light conditions. The elevated calorespirometric ratio under high light conditions provides an indication of photo-protective mechanisms. The Photo-CR delivers the PE in real time, depending on the light intensity. Energetic differences less than 0.14% at radiation densities of up to 800 μE m^−2^ s^−1^ can be quantified. Other photosynthetic growth parameters (e.g. the specific growth rate of 0.071 h^−1^, the cell specific energy conservation of 30.9 ± 1.3 pW cell^−1^ at 150 µE m^−2^ s^−1^ and the number of photons (86.8) required to fix one molecule of CO_2_) can easily be derived from the Photo-CR data.

## Introduction

Photosynthesis is the unique biological process using solar energy and water to convert CO_2_ into organic matter. Understanding and improving the efficiency of this process is an important challenge in green biotechnology and agriculture. For instance, microalgae are considered due to their fast photosynthetic growth rates as promising sustainable feed stock for numerous bioproducts^1,2^. In these applications, it is assumed that a high photosynthetic efficiency will support a positive net balance of energy stored in the algal biomass versus the energy expended for the biotechnological process (e.g. harvest and refinement of algal biomass)^3,4^. In the following the photosynthetic efficiency (PE) is defined as the fraction of light energy converted into chemical energy of biomass during photosynthesis. Several indirect methods are available for determining or estimating PE. The simplest method bases on the stoichiometry of the photosynthesis. The chemically fixed energy can be calculated via the law of Hess based on measurements of the fixed CO_2_ or the formed products such as O_2_, biomass etc. Such investigations are typically carried out in a photobioreactor with the weaknesses of i) an only indirect quantification of the PE, ii) a poor temporal resolution (in the range of a few tens of minutes to hours) and iii) the requirement of considerable manual labor^5^.

In contrast, measurement methods with high temporal resolution, for instance Chl a-invivo fluorometry quantify the energy lost due to non-photochemical quenching (NPQ), but do not consider the energy losses in the subsequent steps of photosynthesis^6–8^. Photoacoustic methods are powerful in delivering a signal proportional to the energy-storage of photosynthesis, but this is only a relative measure of the energy lost into heat^9^. Due to the applied Laser pulse technique only short-term effects can be quantified. For short term measurements, the photothermal beam deflection technique was developed additionally^10^.

Photocalorimetry is the only direct measurement method for PE. First attempts to measure the PE by calorimetry reach back to the late 1930’s^11^. Inspired by the reinvention of photocalorimetry by Wadsö^12^ and others a new impetus came in the photocalorimetric research at the beginning of our century. Photocalorimeters were designed for sample sizes at the mL scale on the basis of isothermal microcalorimetry^8,13^ or at the liter scale on the basis of reaction calorimetry^14–16^. These pioneering calorimetric methods already allowed a limited quantitative determination of the fixed energy. It is all the more surprising that until today, there are hardly any applications for these methods. One reason is the usually low PE of 2–6%^17,18^. The lost energy is mainly dissipated as heat and to a small fraction (1–2%) re-emitted as chlorophyll fluorescence at longer (redder) wavelengths. This extremely challenges the accuracy of calorimetric instruments. Those requirements on the accuracy of the calorimetric device become even more demanding, if energetic differences due to product formation (e.g. hydrogen) or changes in the macromolecular composition of the algal biomass (e.g. lipid accumulation) during the photosynthetic process and their influence on the PE are monitored. The second reason is a challenge in closing energy balances. Energy balances are a common way to interpret calorimetric results. The big challenge here is the precise quantification of the light energy input, the determination of the fraction of absorbed light and of the non-thermal energy output (e.g. biomass, products). The third reason for the lack of application of photocalorimetry is the difficulty to follow dynamic processes during photosynthesis for instance dynamic changes in the quantum yield during an illumination period by the state-of-the-art technologies. However, especially those dynamics are of high interest to study the response of PE in dependency on the environmental conditions. The fourth hurdle is that the measured heat can be influenced not only by the desired energy conservation but also by interfering factors. Here another on-line signal would be useful to avoid misinterpretations, but that is often missing.

These shortcomings call for a new concept, which is called photocalorespirometry (Photo-CR). It provides the direct measurement of PE (calorimetry) in combination with the online measurement of oxygen production (respirometry). The ratio of the energy fixation rate to the oxygen evolution rate is for carbohydrates a constant of approx. −460 kJ mol^−1^. Measured deviations from this calorespirometric ratio may potentially reveal changes in the biomass composition or indicate to the formation of side-products or of other oxygen species such as peroxides^19,20^. The new Photo-CR concept permits the real-time monitoring of dynamic adaptation processes. Furthermore, biases of the PE measurement by e.g. sedimentations and self-shading are excluded. As model organism for the development and testing of our Photo-CR concept the industrially and ecologically important alga *Chlamydomonas reinhardtii* is used.

## Results

### Measuring principle

**Figure 1:**
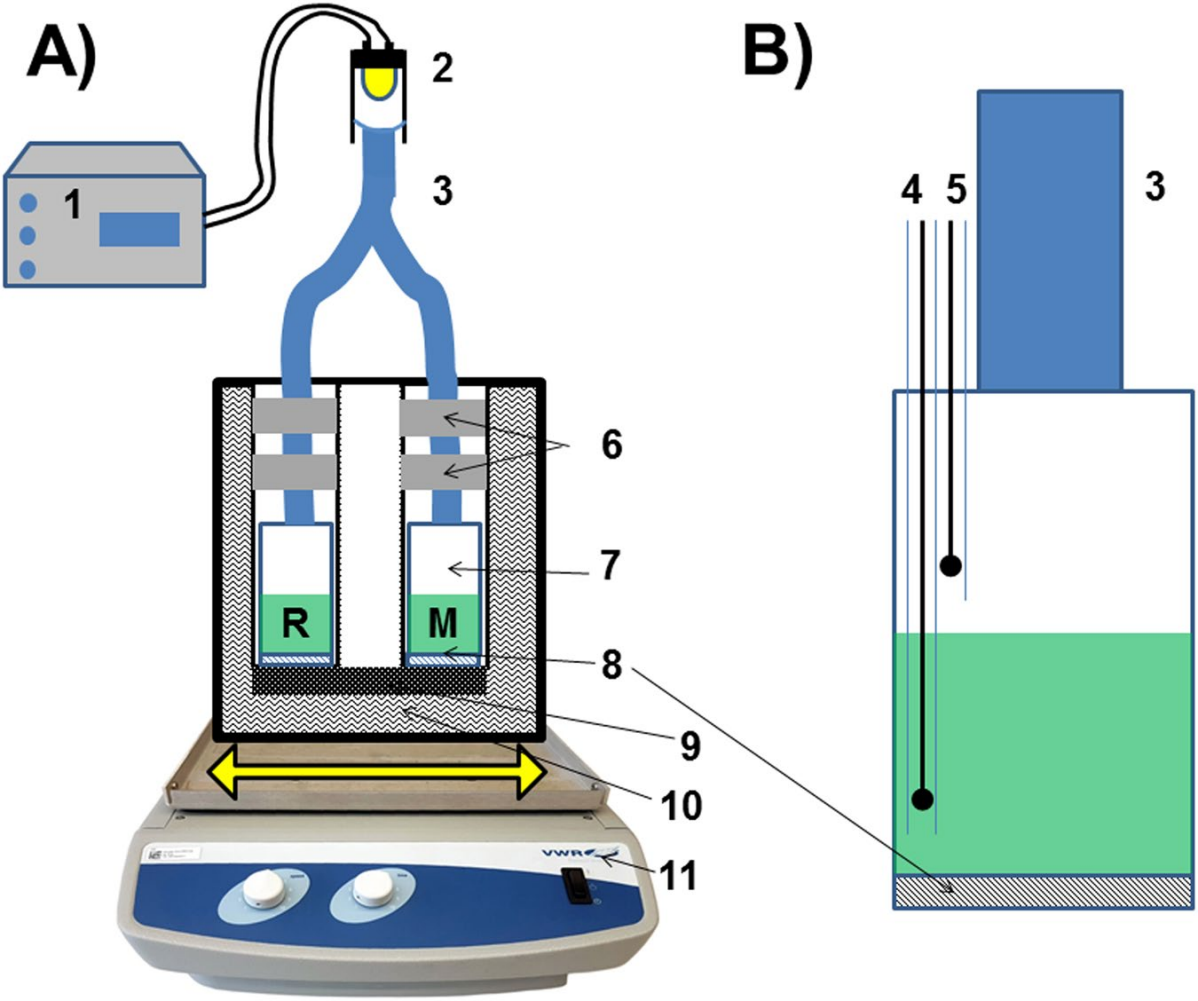
Photocalorespirometric (Photo-CR) setup; (**A**) shows the complete system and (**B**) shows a section through a calorimetric vessel; 1 – Controller of the high-power LED; 2 – LED (light source); 3 – two-arm light guide; 4, 5 – pO_2_ oxygen sensors for the algae suspension and the adjacent atmosphere; 6 – aluminum rings for heat exchange; 7 – calorimetric vessel; 8 – Peltier-elements; 9 – heat sink; 10 – tempered water bath; 11 – horizontal shaker.

The design of the new Photo-CR concept is outlined in Fig. 1. For a better understanding of the following text, all the symbols used and their units are summarized at the end of the methods section. Photosynthetic efficiency (PE, J/J) is the ratio of photosynthetically fixed energy *E*_*PS*_ to the light energy input *E*_*I*_ (eq. 1). The same relationship applies when energy flow rates (*P*_*PS*_ for the fixed energy flow or *P*_*I*_ for the energy input flow; both in W) are used instead of the energies because 1·J = 1·W·s.

1 $$PE=\frac{{E}_{PS}}{{E}_{I}}=\frac{{P}_{PS}}{{P}_{I}}$$

The photosynthetically fixed light energy flow *P*_*PS*_ results from the energy balance around the calorimetric vessel (eq. 2) which includes the heat flow over the Peltier-elements *P*_*Heat*_ (all in W).

2 $${P}_{PS}={P}_{I}-{P}_{Heat}$$

*P*_*Heat*_ is measurable as a voltage output of the Peltier-element. *P*_*I*_ (the light energy input) is regulated by a controlled LED and is known from calibration measurements (Supplementary material, Fig. S2). The light source for the photocalorimeter is a high-power LED whose intensity is adjusted via a voltage controlled LED current driver. The light emitted by the LED is fed into a two-arm light guide. The two arms of that light guide were fixed and thermally equilibrated by aluminum rings.

Additionally to the photosynthetically fixed energy, the oxygen concentration was measured both, in the algal suspension as well as in the head space above, by optical oxygen sensors. Advantageously, optical oxygen sensors work without oxygen reduction and the respective oxidation heat. The entire Photo-CR setup was mounted on a horizontal shaker for mixing and to counteract gradients of nutrients and dissolved gases due to photosynthetic activities or sedimentations of the algae. Additionally, the algal suspension was mixed with 5% Percoll^®^ to avoid sedimentation by increasing the density of the algal suspension. Percoll^®^ was tested before, showing to have no negative effects on the photosynthetic metabolism in the concentrations used (Fig. S8 in Supplementary Material (SM)).

The flux of light energy *P*_*I*_ entering the calorimetric vessel can be calculated using eq. 3.

3 $${P}_{I}={\rm{\Phi }}\cdot {N}_{A}\cdot {E}_{P}\,{\rm{with}}\,{E}_{P}=h\frac{c}{\lambda }$$

Here stands *Φ, N*_*A*_*, E*_*P*_ for the radiation flow (E s^−1^), Avogadro’s number (6.02214 · 10^23^ mol^−1^) and the energy per photon (J), respectively. The energy per photon *E*_*P*_ depends on speed of light in vacuum *c* (2.998 · 10^8^ m s^−1^), the Planck constant *h* (6.62608 · 10^−34^ J s) and the wavelength *λ* (m). We used blue LED with a narrow light spectrum around *λ* = 455 ± *18 nm*. The *in-vivo* absorption spectrum of *Chlamydomas reinhardtii* is given in the supplementary material SM (Fig. S3) to demonstrate the spectral overlap between the cell absorption and the light source. *E*_*P*_ can be calculated thereof to be 4.366 · 10^−19^ J. From the characterization of the LED/light guide system (see SM, Fig. S3) incident light energy (*P*_*I*_) at the surface of the algal suspension of 19.58 mW (100 mA); 61.15 mW (300 mA); 105.9 mW (500 mA) was measured, respectively (Table 1).

**Table 1:** Information about the irradiance conditions and the maximum error of the calorimetric measurements.

LED driver current (*I*_*LED*_) mA	Radiation flow (Φ) µE s^−1^	Irradiance at the surface of algal suspension µE m^−2^ s^−1^	Incident light energy (*P*_*i*_) µW	Number of measurements	Standard deviation (${{\boldsymbol{\sigma }}}_{{{\boldsymbol{P}}}_{{\boldsymbol{i}}}}$) µW	Relative Standard deviation %
100	0.074	150.2	19600	19	25	0.13
300	0.232	469.2	61100	10	73	0.12
500	0.402	795.4	105800	6	45	0.04

The light from the LED is distributed approximately uniformly by the two arm light guide on a measuring (M) and reference (R) side. However, the energy balance (eq. 2) has now to consider the division of the light into the M and R side. That can be achieved by measuring the heat flow difference ${\rm{\Delta }}{P}_{Heat}^{Dead}$ between the M and R side if both sides are filled with photosynthetically inactive material (eq. 4). The use of an algae suspension that was metabolically inactivated with 0.2% formaldehyde proved to be particularly advantageous.

4 $${\rm{\Delta }}{P}_{Heat}^{Dead}={P}_{I}^{M}-{P}_{I}^{R}$$

If the M side is filled with active algae and the R side by killed algae than ${\rm{\Delta }}{P}_{Heat}^{Live}$ is measured and *P*_*PS*_ can be calculated using eq. 5.

5 $${P}_{PS}={\rm{\Delta }}{P}_{Heat}^{Live}-{\rm{\Delta }}{P}_{Heat}^{Dead}$$

Equation 5 assumes that the light and measuring conditions do not change between the two measurements (${\rm{\Delta }}{P}_{Heat}^{Dead}$ and ${\rm{\Delta }}{P}_{Heat}^{Live}$). The better this assumption is fulfilled, the lower the measurement error.

### Reproducibility

**Figure 2:**
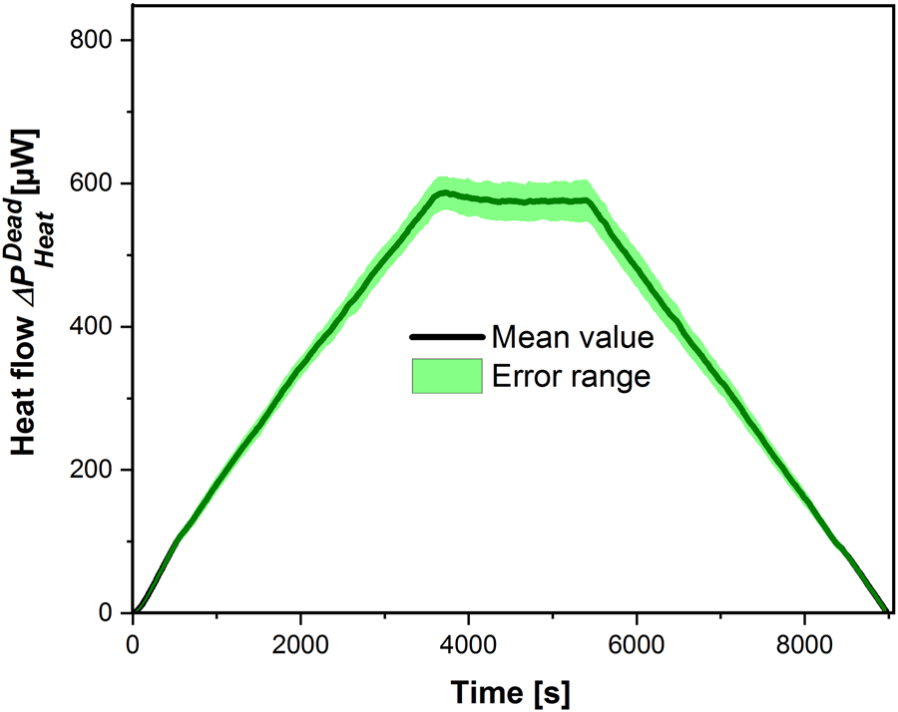
Reproducibility of 19 heat flow measurements of a suspension of inactivated *Chlamydomonas reinhardtii* 11/32B ((7.39 ± 0.22)·10^5^ cells mL^−1^, treated with formaldehyde). The incident radiation energy follows a trapezoidal ramp with ${P}_{I}^{Max}$ = 19.6 mW. The error range is given as mean value ± standard deviation (n = 19). The minimum and maximum values were 542.7 µW and 632.5 µW, respectively.

Because PE is in the single-digit percentage range and the difference between two measurements (${\rm{\Delta }}{P}_{Heat}^{Dead}$ and ${\rm{\Delta }}{P}_{Heat}^{Live}$) has to be evaluated, the accuracy and the reproducibility of the calorimetric measuring system are crucial. The reproducibility of the measurement of ${\rm{\Delta }}{P}_{Heat}^{Dead}$ is shown in Fig. 2 (19 experiments: 4 mixtures with inactivated algae with 4 – 5 repeated measurements each) for a radiation following a trapezoidal ramp of the LED driver current (${I}_{LED}^{Max}=100\,mA;\,{P}_{I}^{Max\,}=19.6$ mW). The corresponding maximum irradiance at the surface is depicted in Table 1.

For all 19 measurements (3420 data points) at the plateau ${\sigma }_{{P}_{i}}$ of 25 µW was measured. The measurement error of living cells ${\rm{\Delta }}{P}_{Heat}^{Live}$ is in the same order of magnitude as Figure S6 in SM demonstrate. The relative error (0.13%) refers to the energy input *P*_*I*_ (19.6 mW at the plateau). The experiments at higher radiations provided higher absolute but lower relative standard deviations. These values are summarized in Table 1.

### Photocalorespirometric characterization of photosynthesis

**Figure 3:**
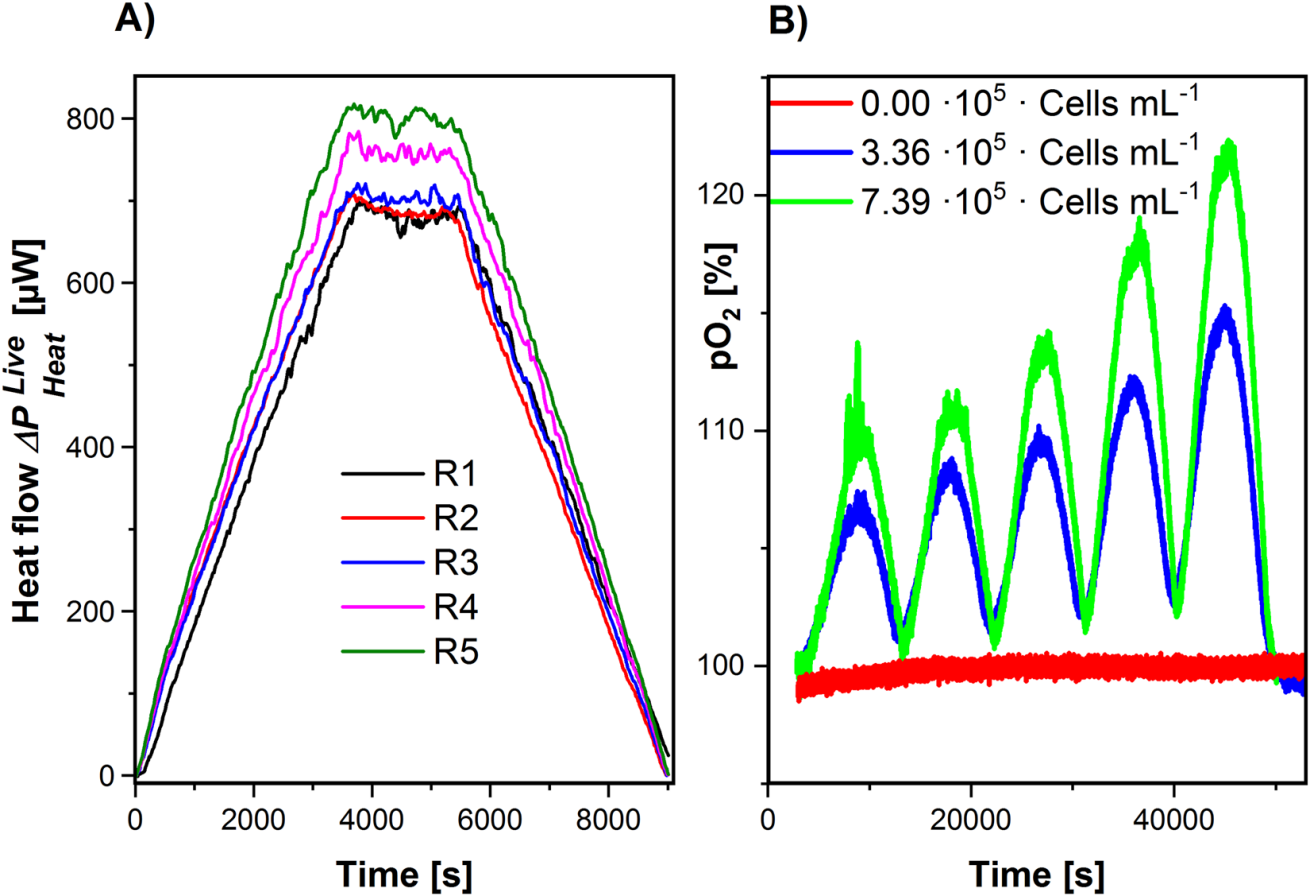
Photocalorespirometric pattern of an algal suspension of *Chlamydomonas reinhardtii* 11/32B responding on a five-times repeated radiation ramp (R1 to R5) with ${P}_{I}^{Max}$ = 19.6 mW. (**A**) Shows the heat flow ${\rm{\Delta }}{P}_{Heat}^{Live}$ for (7.39 ± 0.22)·10^5^ cells mL^−1^ and (**B**) shows the measured oxygen evolution of two different biomass concentrations and the reference (medium without algae). The R numbers in (**A**) count the consecutive repetitions of the ramps.

The peculiarity of our Photo-CR approach is the simultaneous monitoring of PE and the oxygen production. Typical raw data is shown in Fig. 3. For the sake of clarity in Fig. 3, the heat traces are superimposed while the oxygen signal is continuously displayed. Figure 3B additionally shows the oxygen traces with different algae concentrations and with the culture medium (without algae). The heat signal ${\rm{\Delta }}{P}_{Heat}^{Live}$ is larger as with dead cells ${\rm{\Delta }}{P}_{Heat}^{Dead}$. ${\rm{\Delta }}{P}_{Heat}^{Live}$ is increasing over time due to algal growth indicating that photosynthesis is governing the signal. The oxygen signal also grew over time. However, it is noticeable that the oxygen signal does not follow the trapezoidal shape of the light input. The reason is that the current oxygen accumulation rate in the algal suspension *dO*^*L*^*/dt* (mol L^−1^ s^−1^) and consequently also the oxygen concentration *O*^*L*^ (mol L^−1^) is determined by two factors: i) the algal oxygen production rate *r*_*O2*_ (mol L^−1^ s^−1^) and ii) the oxygen loss rate ${k}_{V}\cdot ({O}^{L}-{k}_{H}\cdot {\xi }_{O}^{V}\cdot \pi )$ due to the gas exchange between the algal suspension and the gas phase within the ampoule. The parameter *k*_*V*_ (s^−1^) describes the gas exchange. *O*^*L*^, *k*_*H*_, ${\xi }_{O}^{V}$ and *π* represent the current oxygen concentration in the algal suspension (mol L^−1^), the Henry coefficient under measurement conditions (1.3·10^−8^ mol L^−1^ Pa^−1^)^21^, the volume fraction of oxygen in the gas phase and the total pressure (Pa), respectively. However, the oxygen production rate can be calculated from the oxygen measurements in the algal suspension (*O*^*L*^) and in the gas phase (${\xi }_{O}^{V}$) under the consideration of the two effects (eq. 6).

6 $${{\rm{r}}}_{O2}=\frac{d{O}^{L}}{d\,t}+{k}_{V}\cdot ({O}^{L}-{k}_{H}\cdot {\xi }_{O}^{V}\cdot \pi )$$

**Figure 4:**
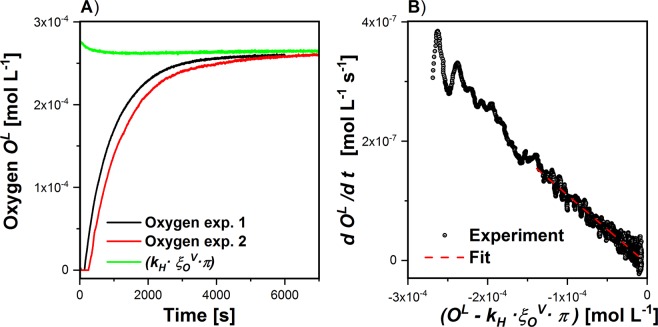
Determination of the gas exchange parameter (*k*_*V*_). (**A**) Shows the oxygen enrichment and the gas transfer from the gas phase. (**B**) Shows the respective parameter fitting $\frac{d{O}^{L}}{d\,t}=-{k}_{V}\cdot ({O}^{L}-{k}_{H}\cdot {\xi }_{O}^{V}\cdot \pi )$.

The accumulation rate (*dO*^*L*^*/dt*) is obtained from the numerical differentiation of *O*^*L*^ after time. The oxygen concentrations in the aqueous phase (*O*^*L*^) were calculated from the percentages of the optode measurements using the well-known oxygen solubility in nutrient solutions^22^. The unknown parameter *k*_*V*_ = *(1.150* ± *0.015)* 10^*−3*^* s*^*−1*^ was determined by gas exchange measurements without algae (Fig. 4). For that purpose, the experiment started with oxygen-free medium and the oxygen uptake from the gas phase was monitored in both the liquid as well as the gas phase. The oxygen-free medium was generated by purging the medium with nitrogen.

### Photocalorespirometry at low light conditions

**Figure 5:**
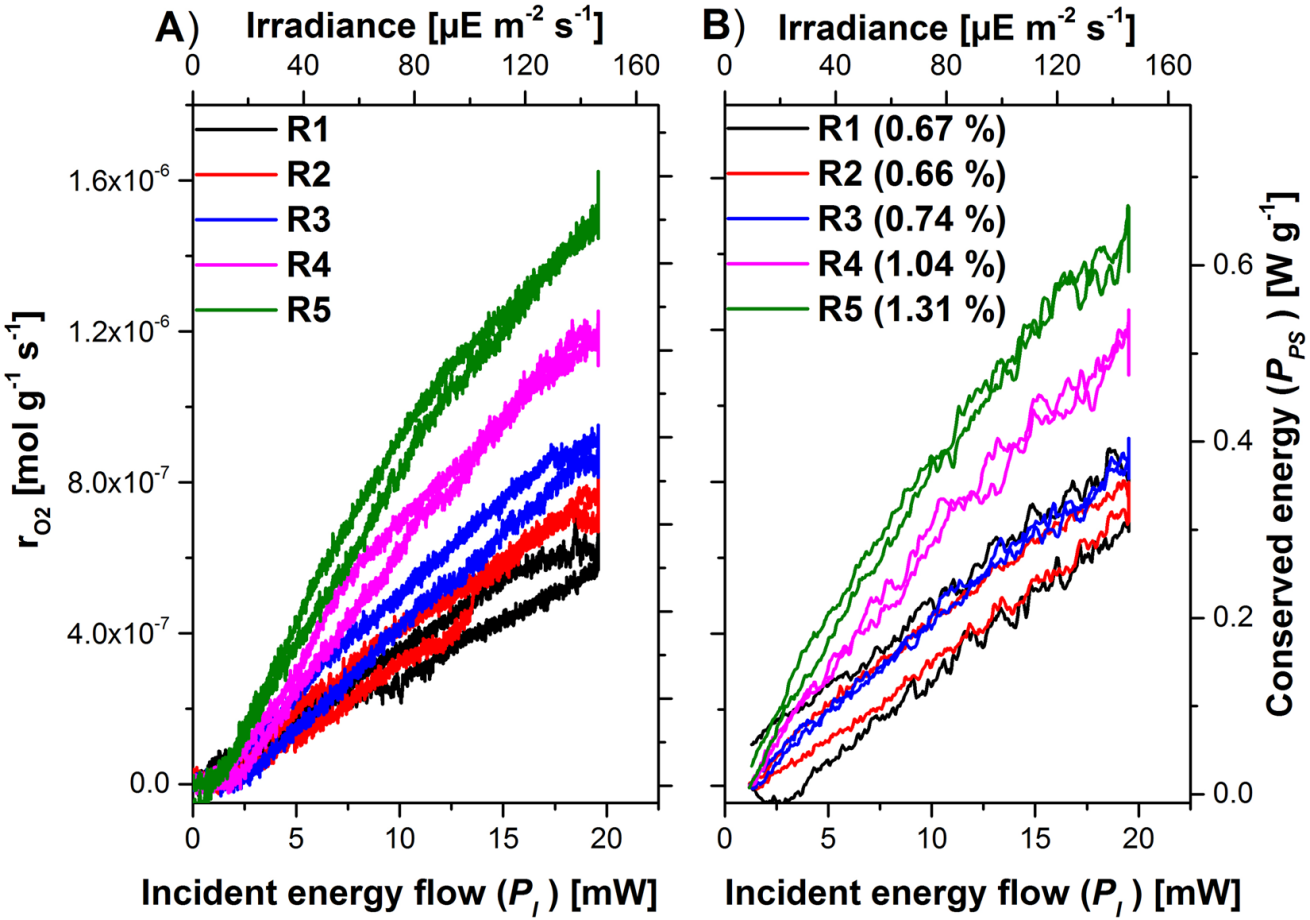
Comparison of oxygen production rate (**A**) with photosynthetically conserved light energy (**B**) as function of light intensity for *Chlamydomonas reinhardtii* 11/32B for low light conditions. Both abscissae were scaled to fulfill the oxycaloric equivalent of −455 kJ mol^−1^. In parenthesis the derived average PE is given. The radiation was five-times repeated with a trapezoidal ramp with ${P}_{I}^{Max}$ = 19.6 mW. The R numbers count the repetitions of the ramps.

**Figure 6:**
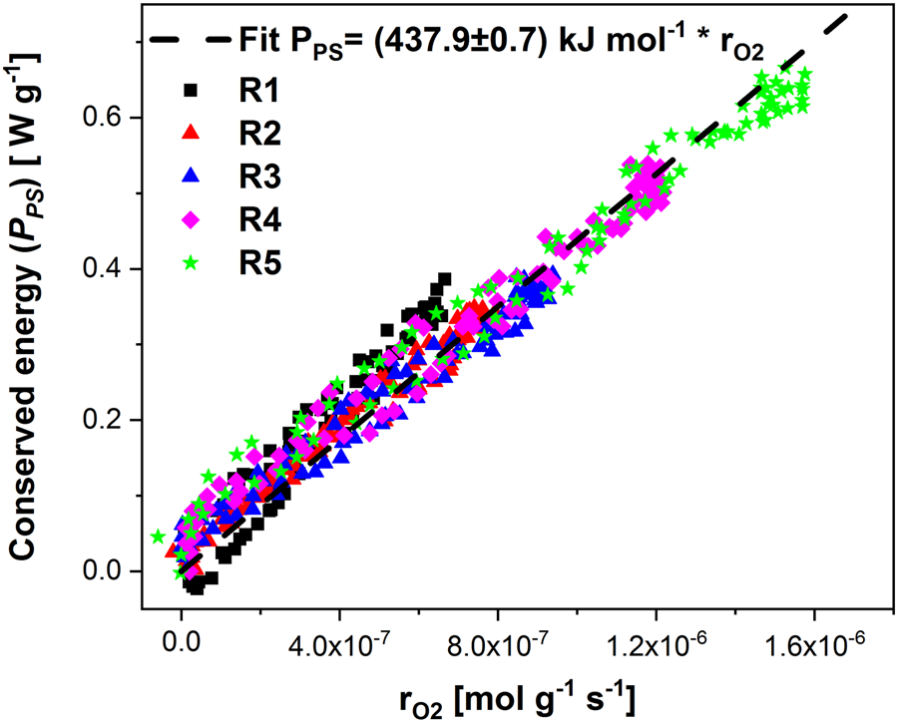
Relation between photosynthetically conserved light energy and oxygen production rate at different light intensities for *Chlamydomonas reinhardtii* 11/32B for low light conditions. Pearson’s correlation coefficient is 0.9937 and the p-value of the t-test for the slope is 0, meaning that the slope is significantly different from zero assuming significance of 95% and there is a strong linear correlation.

This *k*_*v*_ parameter allows the calculation of the algal oxygen formation rate and its comparison with the calorimetrically measured photosynthetically fixed energy flow *P*_*PS*_. For heterotrophic growth a ratio between the heat production rate and the oxygen consumption rate (called oxycaloric equivalent or calorespirometric ratio) in the range (−430 to −480) kJ mol^−1^ for a variety of metabolic substances and growth conditions is described in the literature^23,24^. The application of the oxycaloric equivalent (mean value −455 kJ mol^−1^) allows to scale the oxygen production rate so that it becomes comparable with the energy conservation rate. Figure 5 shows this comparison for low irradiances (*IR* < 150 µE m^−2^ s^−1^). Each point in Fig. 5B) represents the directly measured photosynthetic efficiency (*PE* = *P*_*PS*_/*P*_*I*_) in real time. The average *PE* for the different ramps is given in parenthesis. Assuming a constant stoichiometry for the global photosynthetic process a linear relation between the conserved energy and the produced oxygen is expected. Indeed, such a linear relation with the correlation factor of −(437.9 ± 0.7) kJ mol^−1^ was found (Fig. 6). This factor is in the region of reported oxycaloric equivalents (−430 to −480) kJ mol^−1^)^23^.

### Photocalorespirometric characterization at high light conditions

**Figure 7:**
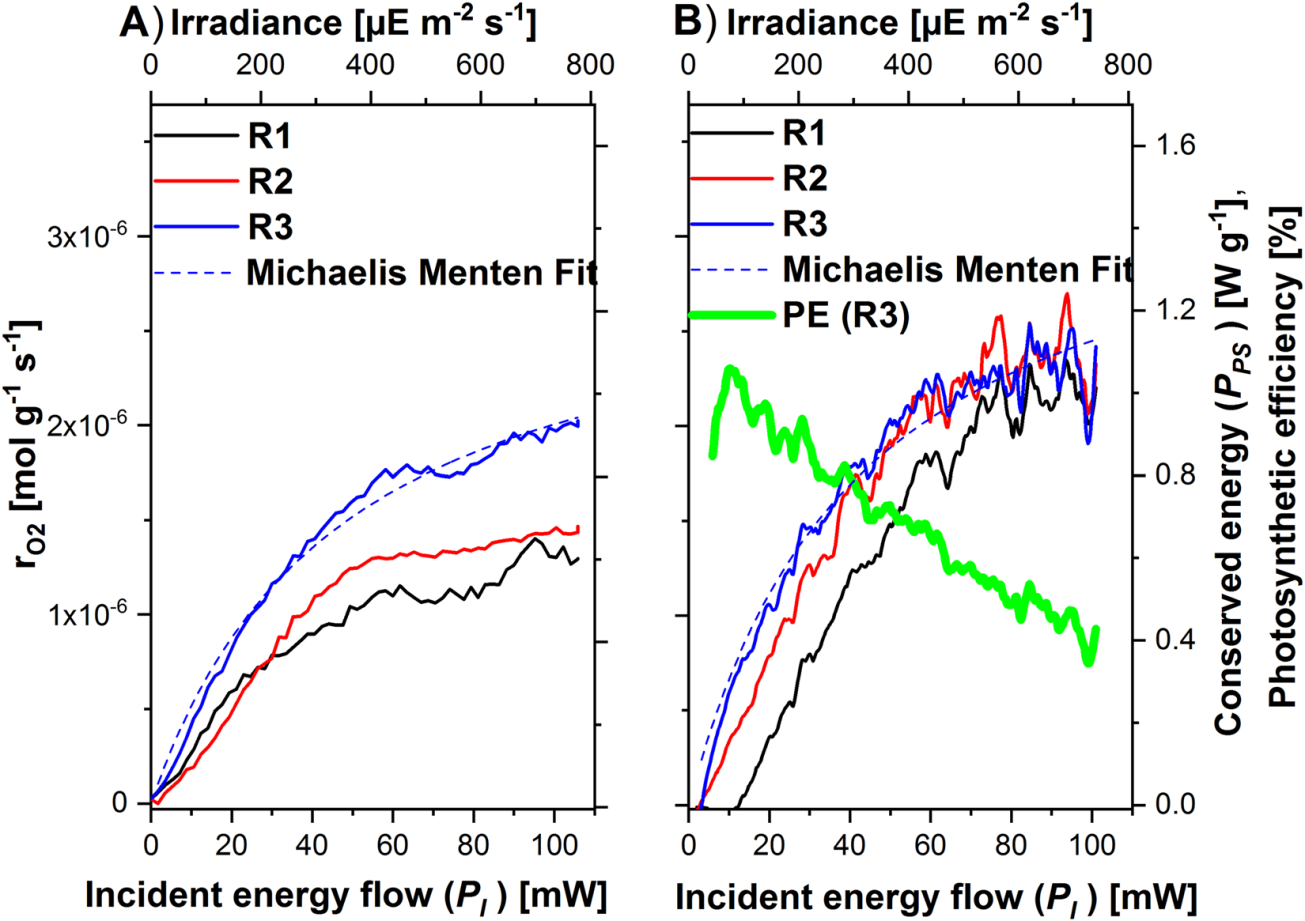
Comparison of oxygen production rate (**A**) with photosynthetically conserved light energy flow (**B**) as function of light intensity for *Chlamydomonas reinhardtii* 11/32B for high irradiances. Both abscissae were scaled to fulfill the oxycaloric equivalent of −455 kJ mol^−1^. (**B**) Shows exemplarily the photosynthetic efficiency (PE) for the third ramp. The irradiation was repeated three-times with a trapezoidal ramp with ${P}_{I}^{Max}$ = 105.8 mW or 712.8 µE m^2^ s^−1^ at the surface of the algal suspension. For clarity, only the curve of the upregulation of the light intensity is shown. The R numbers count the repetitions of the ramps.

It is well known, that under high light conditions photosynthesis rate is reduced. The question thus arises how the Photo-CR displays such the light inhibition and what potential information about the protective mechanisms can be derived from the Photo-CR data. For answering these questions, the oxygen evolution rate and the energy conservation rate was measured under increasing illuminance until light intensity causes saturation. Figure 7 shows the correlation of those measurements.

The comparison reveals that both oxygen evolution as well as the photosynthetically conserved light energy describes the well-known saturation behavior. The PE under high light conditions (Irradiance > 200 µE m^−2^ s^−1^) ceases to be a constant and becomes a function of light intensities. This is shown in Fig. 7B as a curvature of the function *P*_*PS*_ = *P*_*PS*_ *(P*_*I*_). The PE can be determined in real time from the measurement by the quotient PE = *P*_*PS*_*/P*_*I*_ and is shown in Fig. 7B as green line.

### Photocalorespirometric characterization of adaptation to carbon deficiency

**Figure 8:**
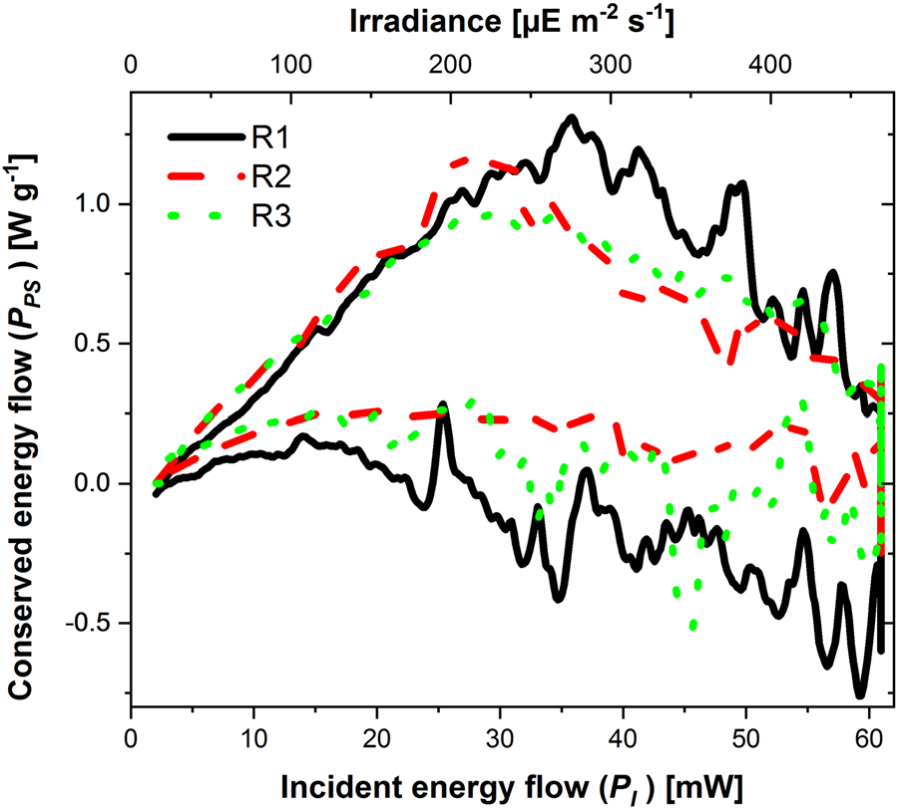
Influence of carbon source deficiency on PE: Heat conservation pattern of *Chlamydomonas reinhardtii* 11/32B during a shortage of the carbon source ((7.39 ± 0.22)·10^5^ cells mL^−1^). The trapezoidal radiation ramp with ramp with ${P}_{I}^{Max}$ = 61.1 mW was three times repeated. The upper part of the hysteresis stands for the upregulation of radiation and the lower part for the down regulation. The R values count the repetitions of the ramps.

To show the potential of the Photo-CR set-up, for monitoring changes of PE under physiological restrictions we performed measurements under CO_2_ limitation. For that purpose, an experiment was conducted with a deficiency (1.5 mM; 15% of the usual amount) of the carbon source. The surprising result is shown by Fig. 8.

For the first irradiation ramp during the upregulation, a similar increase of the photosynthetically fixed energy is observed as in the previous experiments with sufficient CO_2_. However, at *P*_*I*_ = *35 mW* a maximum of *P*_*PS*_ was observed. Thereafter, *P*_*PS*_ decreased continuously presumable due to the shortage of the carbon source. During the steady state radiation phase (*P*_*I*_ = *61.1 mW*) the energy turned from conservation (0.3 W g^−1^) to even energy dissipation (−0.6 W g^−1^). Even during the downregulation of the irradiation, little or no energy is conserved. The second and the third repetition of the irradiation ramp provided qualitatively the same picture. However, the maxima of the conserved energy (R2: *P*_*PS*_ = *1.18* W g^−1^; R3: *P*_*PS*_ = *1.00 W g*^*−1*^) was smaller and was already reached at lower incident energy flow (R2: *P*_*I*_ = *28.5 mW;* R3: *P*_*I*_ = *27 mW*), because a part of the carbon source is already fixed in biomass.

### Characterization of the performance parameters of the Photo-CR

**Figure 9:**
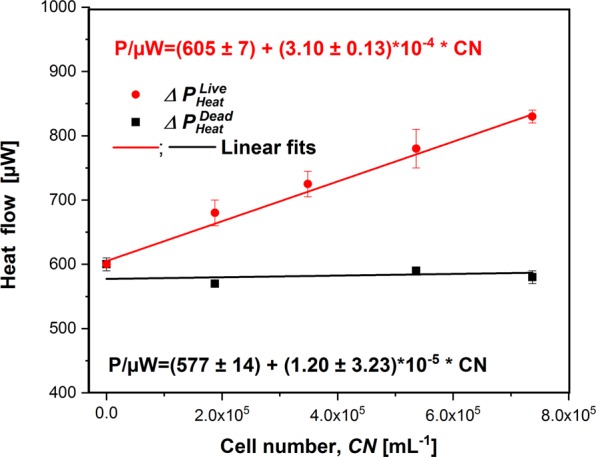
${\rm{\Delta }}{P}_{Heat}^{Live}$ and ${\rm{\Delta }}{P}_{Heat}^{Dead}$ as function of the cell number. The incident light energy was 19.6 mW and the irradiance at the surface 150.2 µE m^−2^ s^−1^. Pearson’s correlation coefficients are 0.997 (live) and 0.273 (dead) indicating a strong linear correlation for living cells and no correlation for dead cells. The p-values of the t-test for living cell are (slope: 1.78·10^−4^; intercept: 2.74·10^−6^) and for dead cells (slope: 0.72; intercept: 5.73·10^−4^) meaning that for living cells intercept and slope and for dead cells only the intercept are significantly different from zero assuming significance of 95%.

For characterization of our Photo-CR approach the lower limit of detection, the cell specific energy conservation and potential restriction of the measurable algae concentration range by effects of self-shading are important. These data were derived by measuring and plotting ${\rm{\Delta }}{P}_{Heat}^{Dead}$ and ${\rm{\Delta }}{P}_{Heat}^{Live}$ versus the biomass concentration (Fig. 9).

Significant effects of self-shading can be excluded from the observed linear relation between ${\rm{\Delta }}{P}_{Heat}^{Live}\,$and biomass concentration. The photosynthetically conserved energy *P*_*PS*_ is obtained as difference between ${\rm{\Delta }}{P}_{Heat}^{Live}$ and ${\rm{\Delta }}{P}_{Heat}^{Dead}$ (eq. 7). Surprisingly, there is even a small difference for cell-free media, which may be due to the light oxidation of traces of the inactivating agent (formaldehyde). This difference is not considered in the following.

7 $${P}_{PS}=(3.09\pm 0.13)\cdot {10}^{-4}\mu W\cdot mL\cdot CN$$

## Discussion

A new concept for the direct monitoring of the photosynthetic efficiency (PE) as function of changing environmental conditions called photocalorespirometry (Photo-CR) was proposed and tested. The method was tested for this proof of the principle in the range of irradiance between 0 and 800 µE m^−2^ s^−1^. The four main advantages of the Photo-CR approach are that i) the PE is measured directly (as ratio of energies), ii) the complete photosynthetic conversion (from the photon to the biomass) is covered, iii) photo-conversions are monitored with a time resolution of a few minutes and iv) the respiration (i.e. the oxygen production) is measured in addition to heat. The importance of the first advantage is obvious taking into account that the PE has so far predominantly been estimated or measured indirectly (e.g. from photosynthetic stoichiometry or from respirometry). Short-term measurements such as fluorescence or photoacoustic represent only the PE of parts of the photosynthetic engine. Growth experiments in photobioreactors have been used to quantify the PE from the photon to biomass^25^. This approach is very time consuming and labor intensive. More important, this traditional approach is not suited to monitor the influence of rapid changes in the environmental conditions on the PE due to the poor time resolution achieved. Our Photo-CR approach overcomes this deficiency. The simultaneous monitoring of photosynthetic conserved energy and oxygen production rate is important. The ratio of the heat flow to the oxygen formation can potentially indicate metabolic pathways^19,20^ and the formation of side products^26^ as demonstrated for heterotrophic growth using calorespirometry. This argument is later deepened.

In order to unlock the full potential of the Photo-CR, there have been and still are a number of difficulties to overcome. The first and most critical point is the accuracy of the PE measurement. The maximum relative error can be estimated to be 21% using eq. 8 (see SM).

8 $$\frac{{\rm{\Delta }}PE}{PE}=2{\rm{\Delta }}\frac{{\rm{\Delta }}{\rm{\Delta }}{P}_{Heat}^{Live}}{{\rm{\Delta }}{P}_{Heat}^{Live}-{\rm{\Delta }}{P}_{Heat}^{Dead}}+\frac{{\rm{\Delta }}{P}_{I}}{{P}_{I}}$$

Equation 8 identifies the difference ${\rm{\Delta }}{P}_{Heat}^{Live}-{\rm{\Delta }}{P}_{Heat}^{Dead}$ and ${\rm{\Delta }}{\rm{\Delta }}{P}_{Heat}^{Live}$ as the main factor for the error. ${\rm{\Delta }}{\rm{\Delta }}{P}_{Heat}^{Live}$ is influenced by biological properties (concentration of the algae and efficiency of their photosynthetic engine) and by device characteristics (i.e. ratio of irradiated thickness to volume ratio of the calorimetric vessel which governs the number of contacts between algae and photons). The algal concentration alone allows a reduction of the error for the factor of three because only one third of the light quants are absorbed in the recent arrangement of Photo-CR (see SM, Fig. S7). A calorimetric chamber with a large irradiated surface and only a thin layer as well as reflective surfaces would allow optimizing the number of contacts between photons and algae. The calorimetric reaction vessel is a closed ampule (see Fig. 1). Therefore, the second crucial source of error in regards of measuring PE is based on the fact that this ampule has to be removed and refilled between measurements of ${\rm{\Delta }}{P}_{Heat}^{Live}$ and ${\rm{\Delta }}{P}_{Heat}^{Dead}$. Hereby the light guide could move slightly and small change in *P*_*I*_ may occur. A flow-through system with a fixed calorimetric cell and optics would reduce significantly that particular error. The actual measurement error in contrast to the above shown maximum error estimation can be derived from the relation between ${\rm{\Delta }}{P}_{Heat}^{Live}$ and ${\rm{\Delta }}{P}_{Heat}^{Dead}$ versus biomass concentration (Fig. 9; eq. 7). After that the relative error in PE is about 4.2%.

If comparing the signal to noise ratio of the oxygen concentration measurement on the one hand and the heat flow detection on the other hand for the recent development stage, then the respirometric part provides slightly better data than the calorimetric part. However, while calorimetry covers the entire reaction space, respirometry measures only the oxygen trace at a particular location of the sensor. This effect was counteracted by shaking the Photo-CR for homogenization and using Percoll^®^ to prevent sedimentation. In order to compare our oxygen measurements with literature data, we will focus on two measurement points (r_O2_ = 6.05 10^−7^ mol g^−1^ s^−1^ at 180 µE m^−2^ s^−1^ and r_O2_ = 13.0 10^−7^ mol g^−1^ s^−1^ at 700 µE m^−2^ s^−1^). The first measuring point can be compared by converting it from a cell dry mass related value to a cell specific value (our measurement: 114 nmol cell^−1^ h^−1^; literature: 77.6 ± 6.1 nmol cell^−1^ h^−1 ^^28^) using the mean cell dry mass (52.5 ± 3.5 pg cell^−1^, calculated from the relations shown in Figs S4 and S5 in SM). The second measuring point can be compared if the oxygen measure is converted from nmol to mg (our measurement: 150 mg g^−1^ h^−1^; literature: 290 mg g^−1^ h^−1 ^^28^). Our oxygen rates seem to be correct, although they do not match the literature values perfectly. Different measuring conditions (illumination, media, homogenization etc.) are probably responsible.

The PE increased from ramp to ramp as biomass concentration increased (Fig. 5). The increase in biomass concentration is also responsible for the observed hysteresis. During the upregulation of the light and during the stationary illumination the algae concentration increased minimally. As a result, the upward curve was slightly shallower than the downward curve. In theory, the difference between the heat curves can be used to extract the specific growth rate *µ* (in h^−1^) from the heat flow or the oxygen production rate using eq. 9.

9 $${X}_{PS}^{R5,\,Max}={X}_{PS}^{R1,\,Max}\cdot exp(\mu \cdot {\rm{\Delta }}t)$$

Here stands ${X}_{PS}^{Ri,\,Max}$ and Δt for the maximum value of a growth parameter of the ramp *i* and the illumination time, respectively. A specific growth rate of 0.071 h^−1^ (from the conserved energy) and of 0.087 h^−1^ (from the oxygen production) was estimated. The value is in the range of literature values between of 0.021 and 0.16 h^−1 ^^27,28^.

From the observed linear correlation between energy fixation and biomass concentration (Fig. 9 and Eq. 7) the cell specific energy conservation can be derived (30.9 ± 1.3 pW cell^−1^) at 150 µE m^−2^ s^−1^. Due to the novelty of our Photo-CR approach, there are no comparative literature data available. A conserved energy of 26.8 ± 0.9 pW cell^−1^ at 180 µE m^−2^ s^−1^ for *Chlamydomonas reinhardtii* can be estimated from the respirometric data of Langner *et al*.^27^ using the oxycaloric equivalent. The slight difference between both values is potentially caused by the applied light source. Another highly important parameter is the number of photons required to fix one mole of carbon dioxide. Light attenuation experiments (Fig. S7 in the SM) show that at the bottom of the calorimetric vessel approximately 40% of the incident light (0.04 × 0.074 µE s^−1^ = 2.96·10^−8^ E s^−1^) is absorbed. Taking the mean value of our rough estimation of the growth rate 0.079 h^−1^ and the biomass concentration at the begin, the biomass formation rate can be estimated to be 8.52 10^−9^ g s^−1^. Thereof the increase of biomass per mol photons can be calculated to be 0.29 g E^−1^. Taking a mean biomass composition of (CH_1.821_O_0.605_N_0.103_)^29^ with a molar mass of 24.9 g mol^−1^, the carbon fixation rate of 3.41·10^−9^ mol s^−1^ results. Thereof (2.96 10^−8^ E s^−1^/3.41·10^−10^ mol-C s^−1^) 86.8 photons per mol fixed CO_2_ results. The minimal theoretical value is 8 photons per mol^30^. Our value of 86.8 photons or 0.29 g E^−1^ is in the magnitude of literature values reported for *Chlamydomonas reinhardtii* (0.293^27^, 0.51–1.25 g E^−1 ^^31^).

The next question was what additional information the Photo-CR provides when adaptation to high light intensities is being investigated. Two different main effects are expected or suspected. First, a saturation curve of *r*_*O2*_ = *r*_*O2*_ *(P*_*I*_) and *P*_*PS*_ = *P*_*PS*_ *(P*_*I*_) is expected and was actually observed. The saturation behavior can be described by a Michaelis-Menten like equation^32^. The obtained parameter are for the third ramp (R3, Fig. 7) *v*_*max*_ = *(1.61* ± 0*.0*2*) W g*^*−1*^ and *k*_*m*_ = *(42.6* ± *1.4) mW* for the conserved energy and *v*_*max*_ = *(2.95* ± 0*.07)·10*^*−6*^*·mol·g*^*−1*^*·s*^*−1*^ (1.36 W g^−1^); *k*_*m*_ = *(47.4* ± *2.5) mW* for the oxygen production rate. The two half saturation parameter *k*_*m*_ are of the same size for both data records. Both values (v_max_ and k_m_) are in the range of literature data (v_max_ = (2.72 ± 0.19)·10^−6^ mol g^−1^ s^−1^ and k_m_ = 331 ± 56 µE m^−2^ s^−1^ (45.1 ± 7.6 mW))^25^. An oxycaloric equivalent (−545,7 ± 20.3 kJ/mol) greater than the literature span for heterotrophic growth between −430 and −480 kJ mol^.−1 24^ is obtained when the v_max_ values are divided. This is an indication for the second presumption, namely a change in the oxycaloric equivalent. The presumption is supported by the observation of a similar deviation in a previous work^33^. At this time, the value could also be attributed to differences in the light conditions between the calorimetric setup and the oxygen measuring device. This ambiguity will be eliminated by our newly developed Photo-CR. We observed for the entire saturation range that the photosynthetically conserved energy was higher as expected from the respirometric measurements assuming −455 kJ mol^−1^ (Fig. 7). A potential metabolic explanation for the observed difference could be the formation of reactive oxygen species (ROS) or of hydrogen peroxide as result of the photorespiration (i.e. glycolate oxidation) or during the Mehler (water/water cycle)^34,35^ under high light conditions. The reaction enthalpy of the Mehler cycle (eq. 10) is endothermic (+98 kJ mol^−1^) analogous to photosynthesis but in contrast to photosynthesis oxygen is consumed. The reaction enthalpy of photosynthesis of glucose (eq. 11) is +2813.6 kJ mol^−1^ but 6 oxygen are formed. This results in a calorespirometric ratio of −469 kJ mol^−1^ in the expected range. Let us assume for illustration purposes, the Mehler cycle runs once per photosynthetic reaction, then the calorespirometric ratio is higher ((2813.6 + 91) kJ/(−5 mol) = −582 kJ mol^−1^) as the literature span according to our observation.

10 $${H}_{2}O+2\,{O}_{2}\to {H}_{2}{O}_{2}+{O}_{2}$$

11 $$6\,C{O}_{2}+12\,{H}_{2}O\to {C}_{6}{H}_{12}{O}_{6}+6\,{O}_{2}+6\,{H}_{2}O$$

Secondly, a reduction in the PE in response to high light conditions was expected and actually observed (Fig. 7). This effect could be caused by photo-protection mechanisms or photorespiration. When photosynthesis is limited by a carbon deficiency, the Photo-CR shows a maximum of the conserved energy versus the incident energy (Fig. 8). With other words, two effects (higher energy input vs. limitation of the photosynthesis) work against each other.

A wide variety of future applications for the Photo-CR are conceivable. The exact knowledge of the PE of phytoplankton in dependency on environmental factors (light irradiation, pH, salinity, dissolved CO_2_, nutrients etc.) is highly important for estimation of the CO_2_ fixation capacity (primary production) of marine ecosystems for instance. After further development of Photo-CR, the technology can be applied to control photobioreactors for stabilization of their performances. Also conceivable are applications in the field of the artificial photosynthesis. But even outside of photosynthetic research is the application for analyzing the processes during a photodynamic therapy^36^ imaginable. Here the photosensitized reactions of triplet oxygen can be analyzed and optimized. Further potential applications outside of biology can be found in photochemistry. Important issues in this context are the stability of pharmaceuticals^37^ and plastics against light, the photopolymerization^38^, and the light-controlled manifold of chemical reactions^39^.

For the next generation of Photo-CR, a wide variety of improvement options should be considered (e.g. application range, measuring throughput, and accuracy). The application range can be extended if light quants of other wavelength (UV or IR) are applied and if the system is equipped with further on-line sensors such as for CO_2_. The design of the Photo-CR as multichannel instrument would further improve the throughput of the measurements. The accuracy of the Photo-CR will significantly improve by designing it as a flow-through instrument. In regards to measuring cell geometry, the goal is to achieve a high surface area to volume ratio which maximizes the contacts between molecules/cells and photons^35,40^. Furthermore, a flow-through system can be connected even at technical scale to photobioreactors.

## Methods

### Algal cultivation

Pre cultures of *Chlamydomonas reinhardtii* 11/32b obtained from the Culture Collection of Algae at Goettingen University (Göttingen, Germany) were grown on shaken Erlenmeyer flasks at 20 °C under permanent illumination with white light (FL40SS W/37, Sanyo) at an irradiance of 3.5 µE m^−2^ s^−1^ for a week. The algae were cultivated on TAP medium. The TAP medium was composed of (in g/L): TRIS BASE (2.42), acetate (1), K_2_HPO_4_ (0.108), KH_2_PO_4_ (0.054), NH_4_CL (0.375), MgSO_4_ • 7H_2_O (0.1), CaCl_2_ • 2H_2_O (0.05). 1 mL per L Hunter’s Trace Elements^41^ was added. The pH was adjusted to 6.5 with KOH. The algae were harvested from exponentially growing cultures (3 days after inoculation).

### The Photo-CR measurements

#### The calorespirometric part of the instrument

The Thermo Activity Monitor (TAM) 2277 (Thermometric AB, Jäfalla, Sweden) at 20 °C was used for the calorimetric measurements. A twin arrangement (two 20 mL identical stainless-steel ampoules for the reference and the test channel, respectively) was removed from the original instrument, transferred into a tempered cylinder 20 ± 0.01 °C (using a thermostat Lauda EcoLine RE 204, Lauda Dr. R. Wobser GmbH & Co. KG, Lauda-Königshofen, Germany) and finally put onto an orbital shaker (Unitwister, UniEquip GmbH, Leipzig, Germany). The ampoules were equipped with two optodes oxygen sensors (for the medium and the gas space; Microx TX3, PreSens Precision Sensing GmbH, Regensburg, Germany). The final setup is sketched by Fig. 1. For each measurement 10 g of algal suspension with 5% Percoll^®^ was transferred in both ampoules, respectively under sterile conditions. Percoll^®^ was used to adjust the density of the medium to the density of the algal cells to suppress sedimentation. KHCO_3_ (10 mM final concentration, or as described in the respective experiment) was added to each sample as inorganic carbon source to avoid or to control CO_2_ limitation. After stabilization of the base line, the cells were three or five times illuminated with trapezoidal ramps of light intensity: (0–3600 s - linear increase from 0 – ${P}_{I}^{Max}$; 3600–5400 s stationary radiation flow of ${P}_{I}^{Max}$; 5400–9000 s linear decrease to 0 µW). ${P}_{I}^{Max}$ is given for each respective experiment. The measurement frequency of the calorimeter was 1 Hz. Formaldehyde (CH_2_O, 1% v/v) was used to completely block any metabolic activity without changes in the absorptivity of the cells to measure ${\rm{\Delta }}{P}_{Heat}^{Dead}$. OriginPro 9 was used for data evaluation.

#### The irradiation part of the instrument

As light source a High-Power LED from ThorLabs (Newton, New Jersey, US) were used. The LED emits blue light at λ = 455 nm with a Full Width at Half Maximum (FWHM) of 18 nm. The maximum allowed current is 1000 mA with an optical power of about 1020 mW. The LED was powered by a ThorLab T-Cube Driver that outputs a constant current limited to 1200 mA (selectable). The current has a ripple of 8 mA @ 570 kHz. The driver is remote controlled via a 12 bit voltage signal (National Instruments USB-6008). This theoretically allows adjusting the LED current by steps of 0.25 mA or 0.25 µW (illumination at the surface of the LED). This value is however limited to 1 mA/mW by the software used with the NI USB-6008. The light of the LED is coupled into a 2-arm flexible light guide (Volpi, 1.5 m length, 8 mm active diameter, Schlieren, Switzerland), splitting the primary light beam into two. The LED and the light guide are adjusted to ensure that the light intensity outputted by both of the light paths is as equal as possible. For quantification of the relation between the irradiance and the applied current (a polynomial 3^rd^ order was found sufficient) a light meter with spherical micro quantum sensor (Walz ULM-500 with US-SQS/L, Ulm, Germany) was used. The quality of the parameter adjustment and of the induced error of the irradiance is shown in Fig. S2B in the supporting material (SM). The dependency of the irradiance *IR* on the surface of the algal suspension (µE m^−2^ s^−1^) on the distance to the light guide *δ* (mm) was measured with the light meter using a hole in a test ampoule. Two parameter exponential functions (eq. 12) were found to represent the data.

12 $$IR=I{R}_{0}+A\cdot (\exp (B\cdot \delta )-1)$$

Figure S2A shows that above a certain distance the light is distributed more or less homogeneously. The used filling volume of 10 mL (δ=22.1 mm) provide a compromise between the necessary sample size and a mainly homogenous light distribution. For calculation of the incident light energy using eq. 1, the incident radiation flow *Φ* (µE s^−1^) has to be known. *Φ* was calculated by dividing *IR*_*0*_ by the area of the optical output of the light guide (*π/4·d*^*2*^). Here represent *d* (m^2^) the diameter of the optical output. The error in the estimation of the incident radiation flow was by this way less than 2% (10–100 mA; 0–100 µE m^−2^ s^−1^) and less than 0.5% ( > 100 mA; > 100 µE m^−2^ s^−1^). Every month as well as after exchange of the LED’s or after changing the arrangement of the components of the Photo-CR the above mentioned measurements were repeated and the functions new adjusted. This new calibration is necessary because of aging of the LED’s and of the light guide.

### Growth parameter

For the calculations and as a reference the cell dry weight *CDW* [g/L] and the cell number *CN* [mL^−1^] are required. The data were obtained from a double determination of the growth parameter (optical density at 682 nm and dry weight determination) as well from the cell counting under the microscope. The optical density at 682 nm was measured immediately after sampling using a double beam spectrophotometer (Hitachi U2000, Scientific Instruments, Schwäbisch Gmünd, Germany). The cell dry weight was determined from 4 ml after centrifugation and oven-drying at 105 °C until weight constancy. The correlation is shown by Figs S4 and S5 in the SM. The following correlations were obtained and used for the calculations.

13 $$CDW=(0.353\pm 0.013)\cdot g/L\cdot OD(682\,nm)$$

14 $$CN=\,(6.72\pm 0.20)\cdot {10}^{6}\cdot cell\,m{L}^{-1}\cdot OD(682\,nm)$$

CDW, CN, OD stands for cell dry weight, cell number and optical density, respectively. Table 2 summarizes all the symbols used and their units.

**Table 2:** Symbols and nomenclature.

Symbol	Meaning	Value	Unit
*A*	Parameter in eq. 11	—	µE m^−2^ s^−1^
*B*	Parameter in eq. 11	—	µE m^−2^ s^−1^
*c*	Velocity of light in vacuum	2.998 10^8^	m s^−1^
*CDW*	Cell dry weight	—	g L^−1^
*CN*	Cell number	—	ml^−1^
*d*	Diameter of the optical output	5.31·10^−4^	m^2^
*E*_*I*_	Light energy input	—	J
*E*_*P*_	Energy per photon	—	J
*E*_*PS*_	Photosynthetically fixed light energy	—	J
*h*	Planck constant	6.62608 10^−34^	J s
*IR*	Irradiance at the surface of the algal suspension	—	µE m^−2^ s^−1^
*IR*_*0*_	Irradiance at the output of the light guide	—	µE m^−2^ s^−1^
*k*_*H*_	Henry coefficient	1.25·10^−8^	mol L^−1^ Pa^−1^
*k*_*V*_	Gas exchange coefficient	—	s^−1^
*N*_*A*_	Avogadro’s number	6.02214 10^23^	mol^−1^
*O* ^*L*^	Oxygen concentration in the algal suspension	—	mol L^−1^
*OD*	Optical density at 682 nm	—	—
*P*_*Heat*_	Heat flow over the Peltier-elements	—	W
*P*_*I*_	Light energy input flow	—	W
${P}_{I}^{Max}$	Maximum light energy input flow	—	W
*P*_*PS*_	Photosynthetically fixed light energy flow	—	W
*PE*	Photosynthetic energy conversion efficiency	—	—
*r*_*O2*_	Algal oxygen production rate	—	mol L^−1^ s^−1^
Greek Symbols			
${\rm{\Delta }}{P}_{Heat}^{Dead}$	Heat flow difference between the M and R side (both sides filled with inactive material)	—	W
${\rm{\Delta }}{P}_{Heat}^{Live}$	Heat flow difference between the M and R side (M side filled with active algae)	—	W
*δ*	Distance between the output of the light guide and the surface of the algal suspension	—	mm
*Φ*	Radiation flow	—	E s^−1^
*λ*	Wave length	—	m
π	Total pressure	—	Pa
${\xi }_{O}^{V}$	Volume fraction of oxygen in the gas phase	—	—

## Supplementary information

Supplementary information
