# Supplementary material for: Photocalorespirometry (Photo-CR): A Novel Method for Access to Photosynthetic Energy Conversion Efficiency
Source: Sci Rep. 2019 Jun 26;9:9298. doi: 10.1038/s41598-019-45296-8 (PMC6594965; doi:10.1038/s41598-019-45296-8)
Supplement: Supplementary file 1 — Supplementary information [file 41598_2019_45296_MOESM1_ESM.pdf]

Supplementary Material

Photocalorespirometry (Photo-CR): A Novel Method for Access to Photosynthetic Energy Conversion Efficiency

Thomas Maskow*[1], Anne Rothe[1], Torsten Jakob[2], Sven Paufler[1], Christian Wilhelm[2]

[1] UFZ - Helmholtz Centre for Environmental Research, Dept. Environmental Microbiology, Leipzig, Permoserstr. 15, D-04318 Leipzig, Germany

[2] University of Leipzig, Biology I, Dept. Plant Physiology, Johannisallee 21-23, D-04103 Leipzig Germany

*Corresponding author:

Thomas Maskow; Phone: +49 341 2351328; e-mail: Thomas.maskow@ufz.de

Table of Contents

1. Quantification of the incident radiation flow 2
2. Light absorption spectrum 4
3. Growth parameters 5
4. Maximum error estimation of the photosynthetic efficiency 6
5. Light attenuation in the calorimetric vessel 8
6. Influence of Percoll® on the growth of *Chlamydomonas reinhardtii* 9

1. Quantification of the incident radiation flow

For balancing the energy in the photocalorespirometer, the amount of light energy entering the calorimetric ampoule is a key value. The experimental set up is shown by (Fig. S1). The energy input can be determined by measuring the irradiance in dependency on the distance to the end of the light guide and on the applied electrical current (Fig. S2).

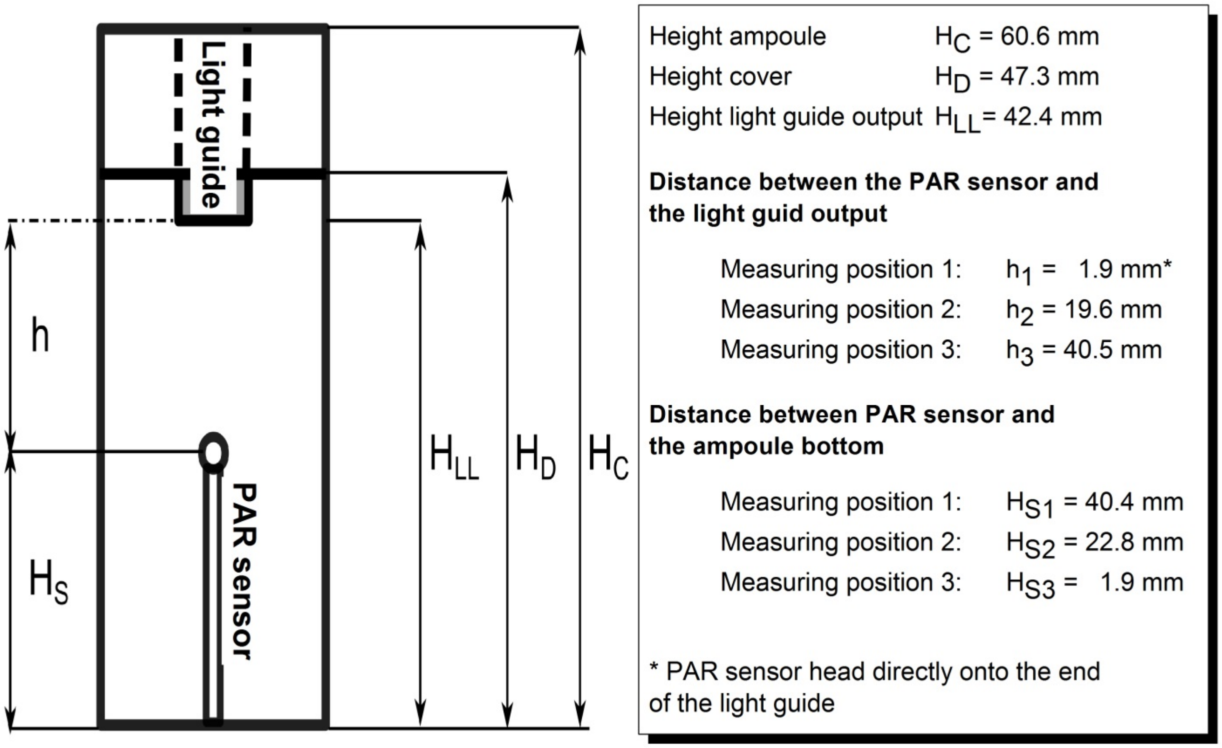

Figure S1: Positioning of the light sensor for calibration and stability tests with the LED lamps

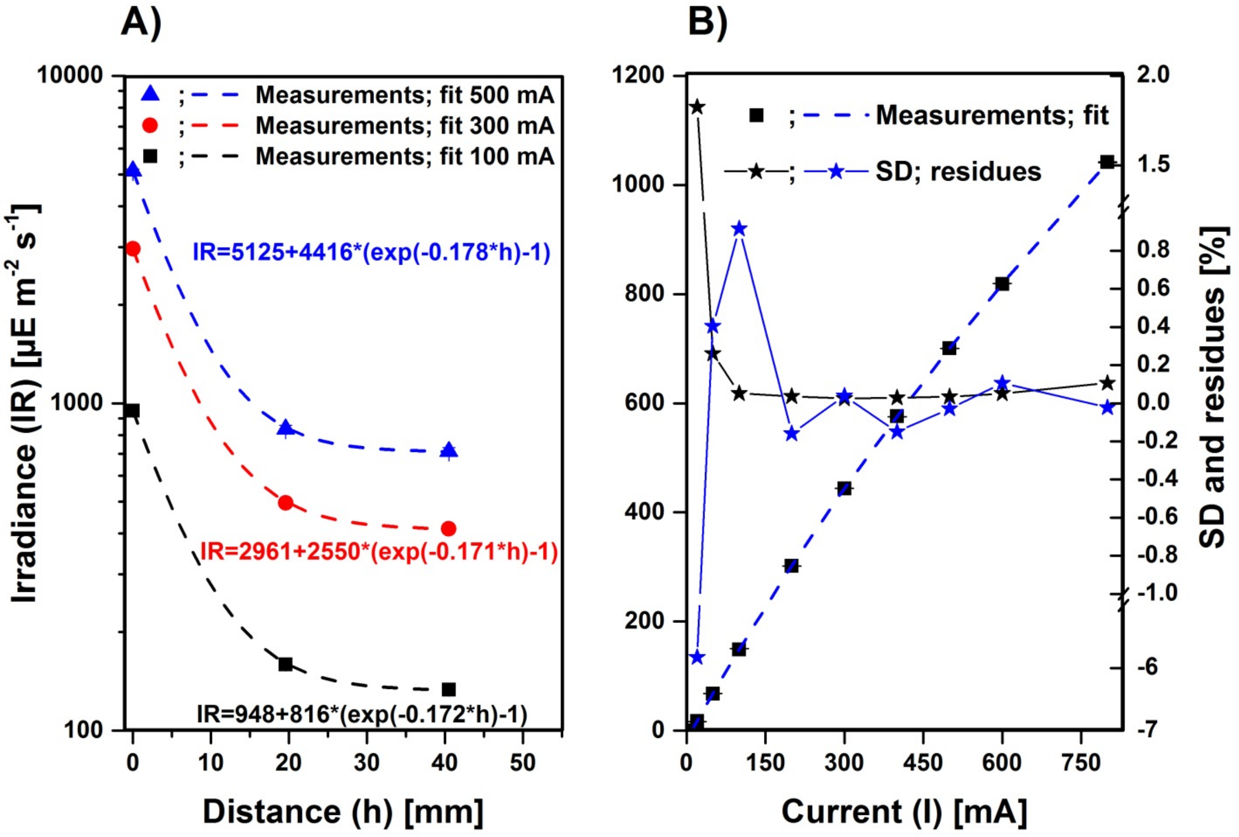

Figure S2: Example of the light calibration of the photocalorespirometer (λ=455 nm). A) Irradiance in dependence on the distance from the light guide. B) Relation between the irradiance and the applied current for h=19.6 mm. The right y-axis illustrates the relative measurement errors (as standard deviation of 1008 measurement points and the residue (difference between the mean value of the measurement and the interpolation using a third order polynomial).

2. Light absorption spectrum

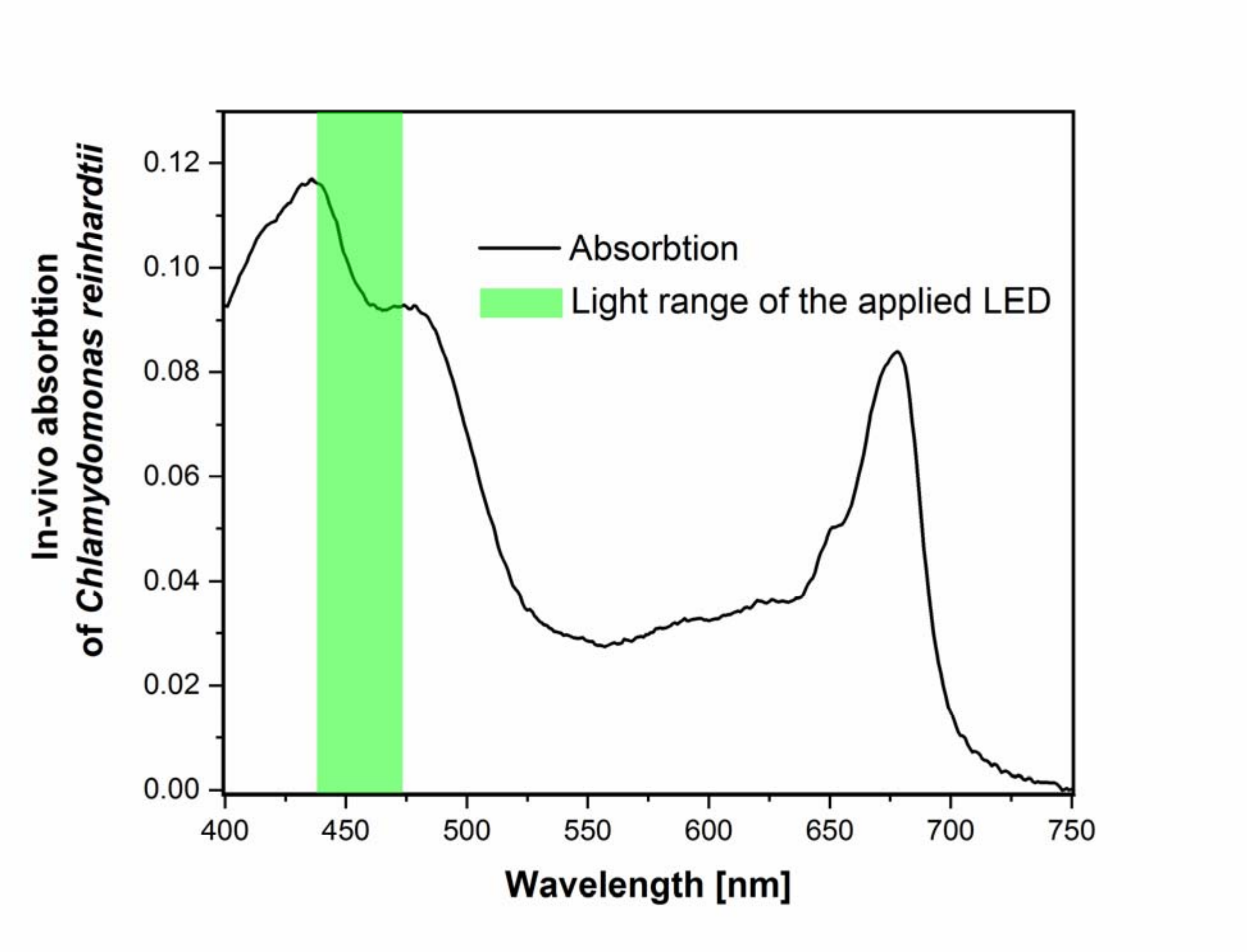

Figure S3: In-vivo light absorption of *Chlamydomonas reinhardtii* and light range of the applied LED.

3. Growth parameters

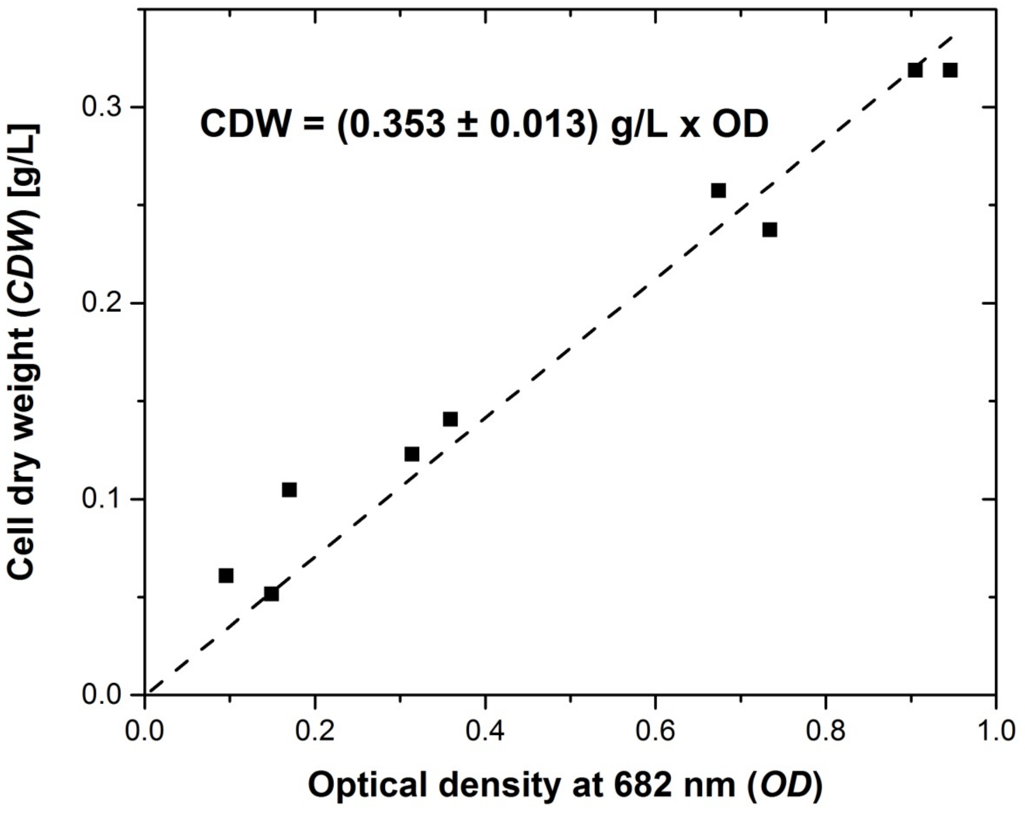

Figure S4: Correlation between the cell dry mass and optical density at 682 nm.

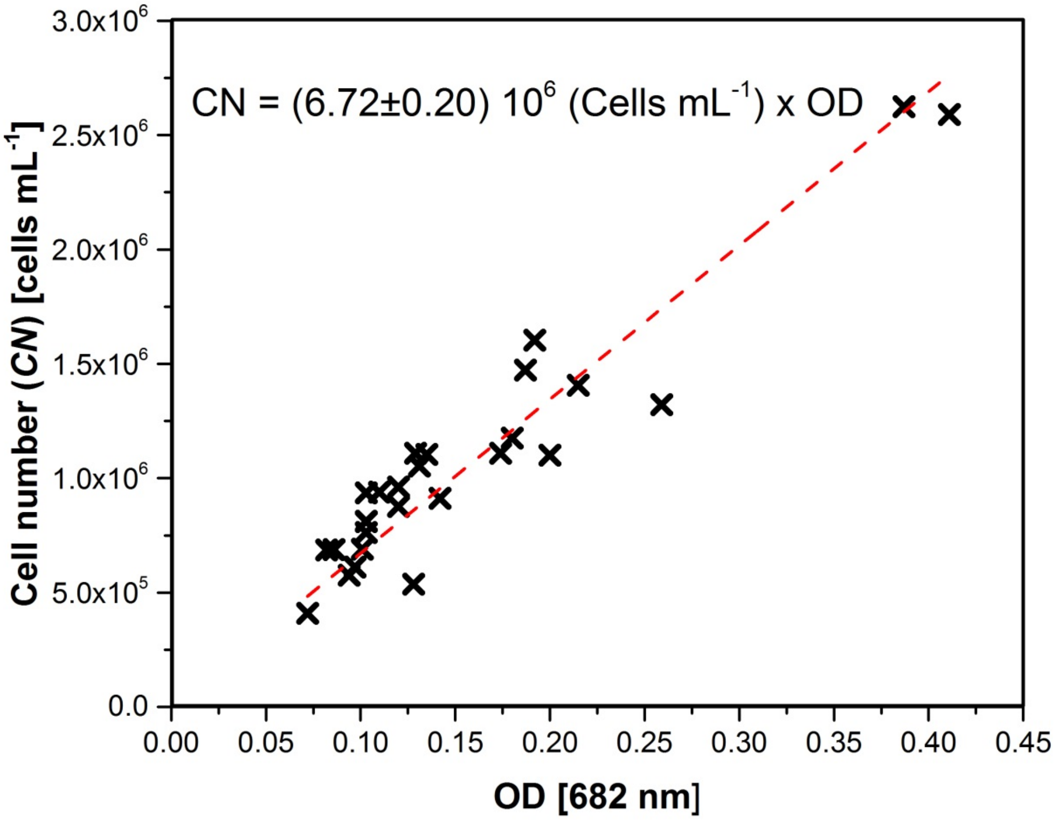

Figure S5: Correlation between the cell number and optical density at 682 nm.

4. Maximum error estimation of the photosynthetic efficiency

The photosynthetic efficiency is calculated according to eq. 1.

$$PE = \frac{\Delta P_{Heat}^{Live} - \Delta P_{Heat}^{Dead}}{P_I} \quad (1)$$

The maximum error can be estimated thereof using eq. 2.

$$\Delta PE = \left|\frac{\partial PE}{\partial \Delta P_{Heat}^{Live}}\right| \cdot \Delta\Delta P_{Heat}^{Live} + \left|\frac{\partial PE}{\partial \Delta P_{Heat}^{Dead}}\right| \cdot \Delta\Delta P_{Heat}^{Dead} + \left|\frac{\partial PE}{\partial P_I}\right| \cdot \Delta P_I \quad (2)$$

$\Delta\Delta P_{Heat}^{Live}$ and of $\Delta\Delta P_{Heat}^{Dead}$ is in the same order of magnitude as figure S6 shows.

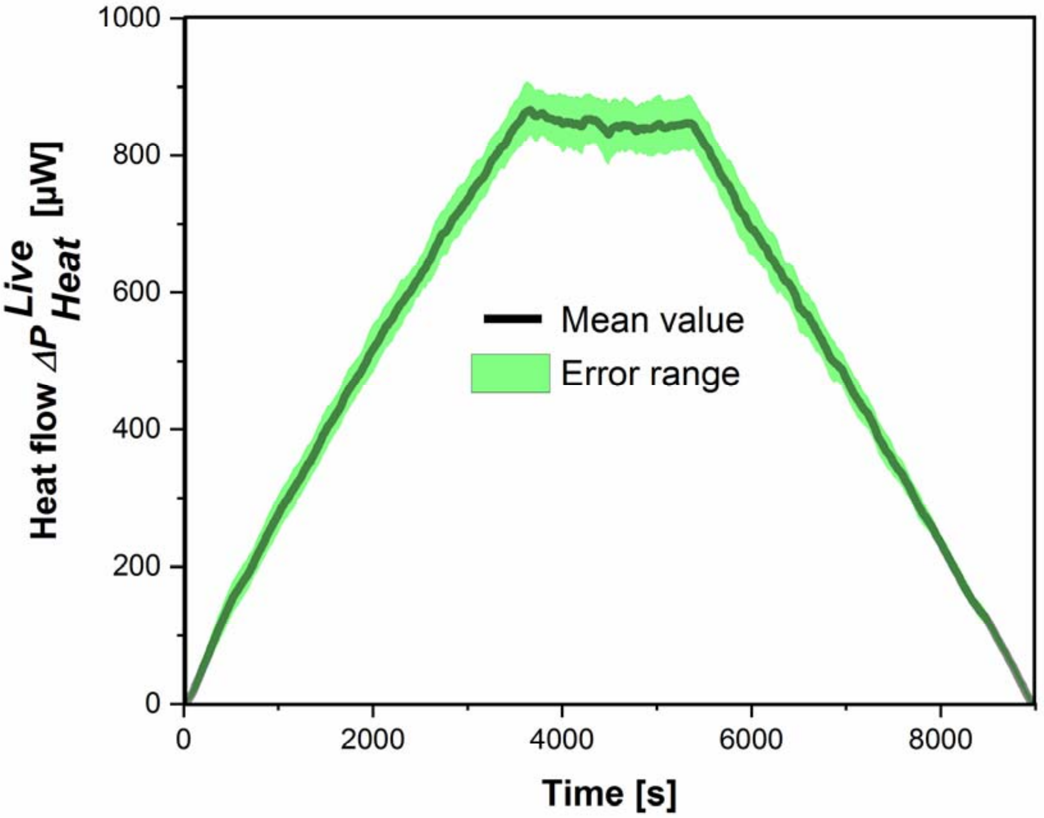

Figure S6: Reproducibility of 6 heat flow measurements of a suspension of active *Chlamydomonas reinhardtii* 11/32B ((7.39±0.22)·10^5 cells mL^{-1}). The incident radiation energy follows a trapezoidal ramp with P_I^{Max} = 19.600 µW. The error range is given as mean value ± standard deviation (n=6).

$$\Delta PE = 2 \cdot \left|\frac{\partial PE}{\partial \Delta P_{Heat}^{Live}}\right| \cdot \Delta\Delta P_{Heat}^{Live} + \left|\frac{\partial PE}{\partial P_I}\right| \cdot \Delta P_I \qquad (3)$$

For the relative maximum error follow from the combination of eq. 1 and 3 the following:

$$\frac{\Delta PE}{PE} = 2 \cdot \frac{\Delta\Delta P_{Heat}^{Live}}{\Delta P_{Heat}^{Live} - \Delta P_{Heat}^{Dead}} + \frac{\Delta P_I}{P_I} \qquad (4)$$

Taking the numbers for the highest radiation: ΔP_{Heat}^{Live} =0.045 mW, $\Delta P_{Heat}^{Live} - \Delta P_{Heat}^{Dead}$=0.427 mW, $\Delta P_I/P_I$=0.002 relative error of approx. 21 % results.

5. Light attenuation in the calorimetric vessel

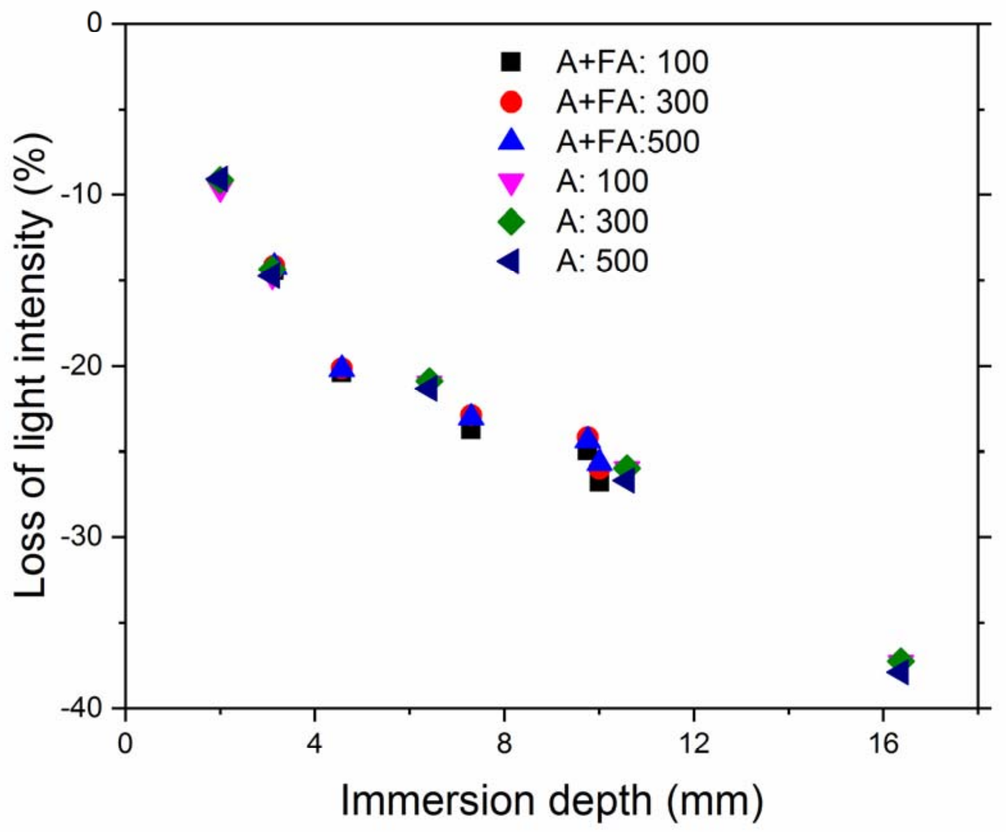

Figure S7: Light attenuation (455 nm) in the calorimetric vessel in dependency on the immersion depth. The numbers stands for the electrical current, driving the LED (for instance 100 - 100 mA). The corresponding irradiance can be taken from table 1 in the main document. A stands for active algae and A+FA for algae poisoned with formaldehyde).

6. Influence of Percoll® on the growth of *Chlamydomonas reinhardtii*

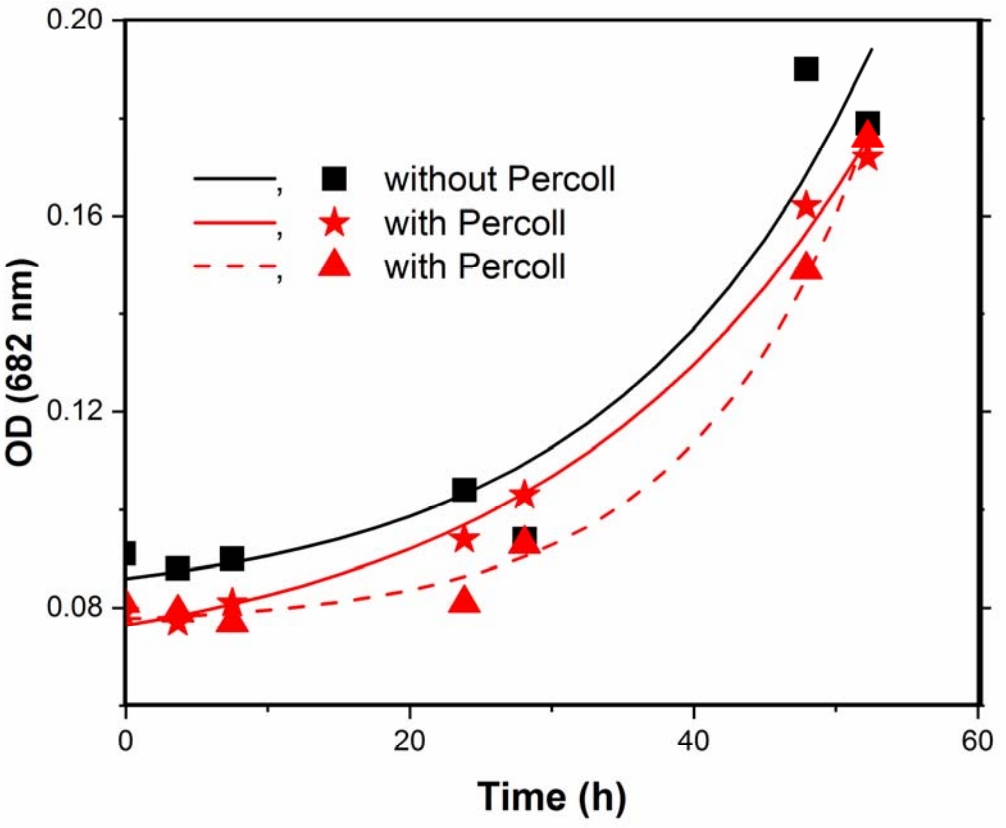

Figure S8: Growth of *Chlamydomonas reinhardtii* in presence and absence of Percoll®.
